# An economic study of neuro-oncological patients in a large developing country: a cost analysis

**DOI:** 10.1055/s-0042-1758649

**Published:** 2022-12-28

**Authors:** Aline Lariessy Campos Paiva, João Luiz Vitorino-Araujo, Renan Maximilian Lovato, Guilherme Henrique Ferreira da Costa, José Carlos Esteves Veiga

**Affiliations:** 1 Santa Casa de São Paulo, Departamento de Cirurgia, São Paulo SP, Brazil.; 2 Hospital do Coração, São Paulo SP, Brazil.; 3 Hospital Sírio-Libanês, Neurosurgery Department, São Paulo SP, Brazil.

**Keywords:** Health Care Costs, Brain Neoplasms, Glioma, Meningioma, Custos de Cuidados de Saúde, Neoplasias Encefálicas, Glioma, Meningioma

## Abstract

**Background** Neuro-oncological patients require specialized medical care. However, the data on the costs incurred for such specialized care in developing countries are currently lacking. These data are relevant for international cooperation.

**Objective** The present study aimed to estimate the direct cost of specialized care for an adult neuro-oncological patient with meningioma or glioma during hospitalization in the largest philanthropic hospital in Latin America.

**Methods** The present observational economic analysis describes the direct cost of care of neuro-oncological patients in Santa Casa de São Paulo, Brazil. Only adult patients with a common primary brain tumor were included.

**Results** Due to differences in the system records, the period analyzed for cost estimation was between December 2016 and December 2019. A group of patients with meningiomas and gliomas was analyzed. The estimated mean cost of neurosurgical hospitalization was US$4,166. The cost of the operating room and intensive care unit represented the largest proportion of the total cost. A total of 17.5% of patients had some type of infection, and 66.67% of these occurred in nonelective procedures. The mortality rate was 12.7% and 92.3% of all deaths occurred in emergency procedures.

**Conclusions** Emergency surgeries were associated with an increased rate of infections and mortality. The findings of the present study could be used by policymakers for resource allocation and to perform economic analyses to establish the value of neurosurgery in achieving global health goals.

## Resumo

**Antecedentes** Pacientes neuro-oncológicos demandam tratamento médico especializado. Em países em desenvolvimento, há falta de dados sobre custos em neurocirurgia. Estes dados são relevantes para ajudar na cooperação internacional.

**Objetivo** O presente estudo objetiva estimar o custo direto de um paciente neuro-oncológico adulto com meningioma ou glioma durante sua internação no maior hospital filantrópico da América Latina.

**Métodos** A presente análise econômica observacional descreve os custos diretos de um paciente neuro-oncológico da Santa Casa de São Paulo. Apenas pacientes adultos e com os dois tumores cerebrais primários mais comuns foram considerados.

**Resultados** Devido a uma mudança no sistema de prontuários, para análise de custos o período analisado foi de dezembro de 2016 a dezembro de 2019. Uma amostra significativa de pacientes com gliomas e meningiomas foi analisada. O custo médio da hospitalização foi de U$ 4.166. O tempo de sala cirúrgica e os cuidados em terapia intensiva representaram a maior proporção dentro do custo total. Um total de 17.5% dos pacientes teve algum tipo de infecção e 66.67% delas ocorreram em procedimentos não eletivos. A taxa de mortalidade foi de 12.5% e 92.3% dos óbitos ocorreram em procedimentos de urgência.

**Conclusões** Cirurgias de urgência foram mais associadas a taxas de infecção e mortalidade. Os achados do presente estudo podem ser usados por planejadores em política pública de saúde para alocação de recursos e para análise econômica para estabelecer o valor dos procedimentos neurocirúrgicos para atingir metas mundiais.

## INTRODUCTION

Five billion people worldwide do not have access to safe and affordable surgical and anesthetic care when needed 1 . Access is worse in low-income and lower-middle-income countries, where 9 out of 10 people cannot access basic surgical care. 1 Neurosurgery in particular is a highly specialized and complex specialty.

Every year, 33 million individuals are affected by catastrophic health expenditure for surgery and anesthesia, and many of these individuals do not have access to medical insurance. Approximately 48 million cases of such excessive expenditure can be attributed to the nonmedical costs of accessing surgical care. 2 3 Furthermore, return to work is challenging after undergoing surgery, and 25% of the people who undergo surgery face a financial crisis. 4 5

Surgical conditions represent 11% of the global burden of disease. 3 Providing surgical care in subspecialties such as neurosurgery is a challenge. Although neurosurgical procedures are less commonly performed compared to those in other specialties, neurosurgical disorders, such as brain tumors, have high rates of morbidity and mortality. 6 Neurosurgery is expensive and requires advanced technology to achieve the best outcomes, especially for brain tumors. The National Institute of Cancer, Brazil, estimated an occurrence of 11,090 new cases of brain tumor every year. 7

Pathological, molecular, and genetic evaluation is required to improve tumor diagnosis, which is essential to guide optimized therapy. 8 9 The most recent World Health Organization (WHO) classification of brain tumors was published in 2021. 5 Patients with primary and secondary brain tumors experience several differences in the treatment, prognosis, and associated costs. Systemic commitment by a primary cancer generally results in an impaired functional status. Thus, surgical outcomes and costs should not be compared between patients with primary and secondary brain tumors.

Meningioma is the most common benign brain tumor, and glioblastoma (GBM) is the most common primary malignant tumor. 1 10 The focus of the present research was to estimate the impact and costs of neurosurgical treatment of patients with gliomas and meningiomas at a large quaternary philanthropic teaching hospital in Brazil.

## METHODS

Cost analysis was performed for a small time period due to changes in the record system of the hospital. The 2016 WHO classification was used to classify the neoplasms. Molecular markers such as H3K27M and are affected by isocitrate dehydrogenase (IDH) were not systematically available for research on the public health system in this hospital. All the gliomas were classified as high-grade or low-grade, or not otherwise specified.

All study procedures were conducted in accordance with the ethical standards of the institutional and national research committee and the 1964 Helsinki Declaration and its later amendments or comparable ethical standards. The present project was approved by the local Institutional Human Ethics Research Committee. Statistical tests were used to analyze quantitative and qualitative variables.

The cost of the entire procedure was determined through a detailed evaluation of the surgical material, operating room, drugs, blood products, parenteral nutrition, imaging examinations, laboratory tests, days of hospitalization, and physiotherapy ( Table 1 ). The value was initially calculated in Brazilian Reais, and later converted to US dollars (this conversion was performed using the US dollar to Brazilian Reais exchange rate, which was 5.37 as of February 06, 2021).

**Table 1:** Variables considered for cost calculation of each procedure

Variable	Mean	Total	%
Operating room	R$ 6.542,51	R$ 673.878,53	29.2
ICU**	R$ 5.582,99	R$ 575.048,10	25.0
Neurosurgical infirmary	R$ 4.140,78	R$ 426.500,10	18.5
Other medications excluding antibiotics	R$ 2.041,17	R$ 208.199,25	9.1
Laboratory, pathology, radiological exams	R$ 1.888,77	R$ 194.543,58	8.4
Specific neurosurgical material	R$ 1.202,78	R$ 121.480,35	5.4
Antibiotics	R$ 324,55	R$ 32.454,93	1.5
Physiotherapy	R$ 180,43	R$ 18.584,39	0.8
Other procedures (gastrostomy, tracheostomy…)	R$ 102,70	R$ 10.578,46	0.5
TOTAL COST	R$ 22.372,22	R$ 2.304.339,07	100.0

Abbreviation: ICU, intensive care Unit

Note: ^*^ using the February 06/2021, exchange rate in which US$ 1 equals R$ 5.37

After the data collection, the free version of R software version 3.3.0 (R Foundation, Vienna, Austria) and IBM SPSS Statistics for Windows version 20 (IBM Corp., Armonk, NY, USA) software were used to perform the statistical analyses. We used the following parameters: mean, median, standard deviation (SD), minimum, and maximum to summarize the quantitative variables. For categorical variables, absolute and relative frequencies (%) were calculated. The Fischer exact test was used to evaluate the association between categorical variables. For quantitative variables, comparison between two groups was done using the unpaired Mann–Whitney U-test, and the nonparametric Kruskal–Wallis test was used to compare more than two groups. The Spearman correlation coefficient was used to evaluate the linear correlation between two quantitative variables.

A literature review about costs incurred in developing countries was performed using the PubMed database.

## RESULTS

After December 2016, the medical record system of the hospital changed and was substantiated with more details. Before this period, the retrospective cost analysis had several biases. Therefore, for cost analysis, we considered only patients who underwent surgery after this change in the medical record system. Furthermore, only adult patients (> 18 years old) were included. Moreover, endoscopic procedures were excluded from the cost evaluation and only microsurgery was considered. The predominant age range was between 46 and 55 years old (23.3%).

**Figure 1:**
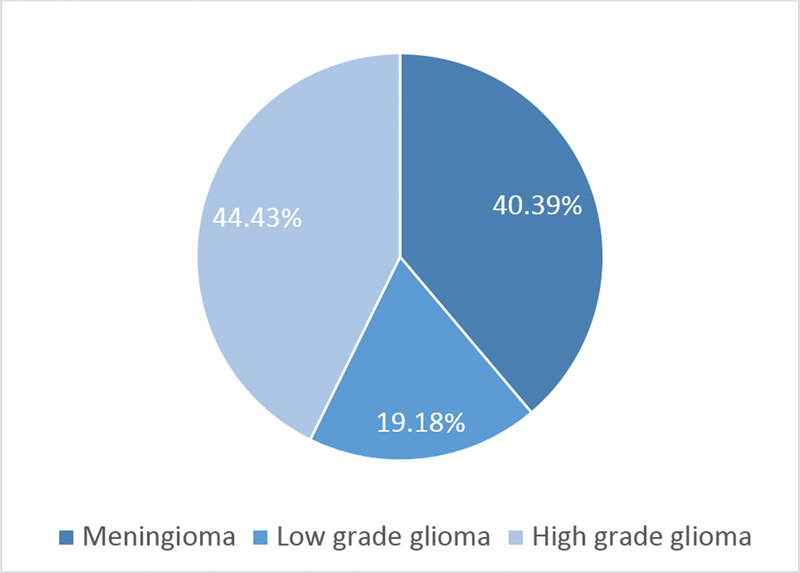
Histological classifications of cost-analysis sample.

The two most common primary tumors were meningiomas and gliomas. A total of 217 patients were included in our study. Considering a margin error of 5%, we calculated the minimum sample size for cost calculation as 139 patients ( *p* < 0.05). To reduce bias, only patients who were operated by neurosurgeons specializing in brain tumors were included. Thirty-seven patients were excluded from the study due to their incomplete data in the hospital records. The cost analysis of hospitalization was performed for 102 patients. Among them, 44.43% had high-grade gliomas, 40.39% had meningiomas, and 19.18% had low-grade gliomas ( Figure 1 ).

**Figure 2:**
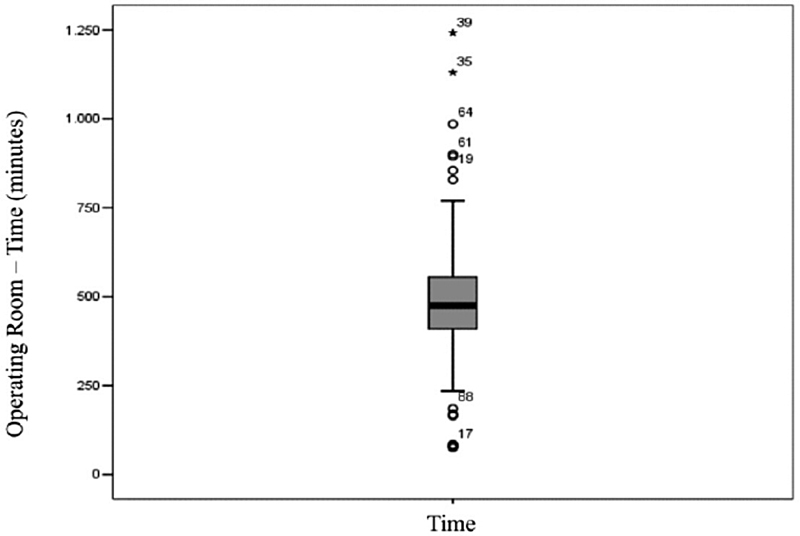
Operating room time in minutes.

**Figure 3:**
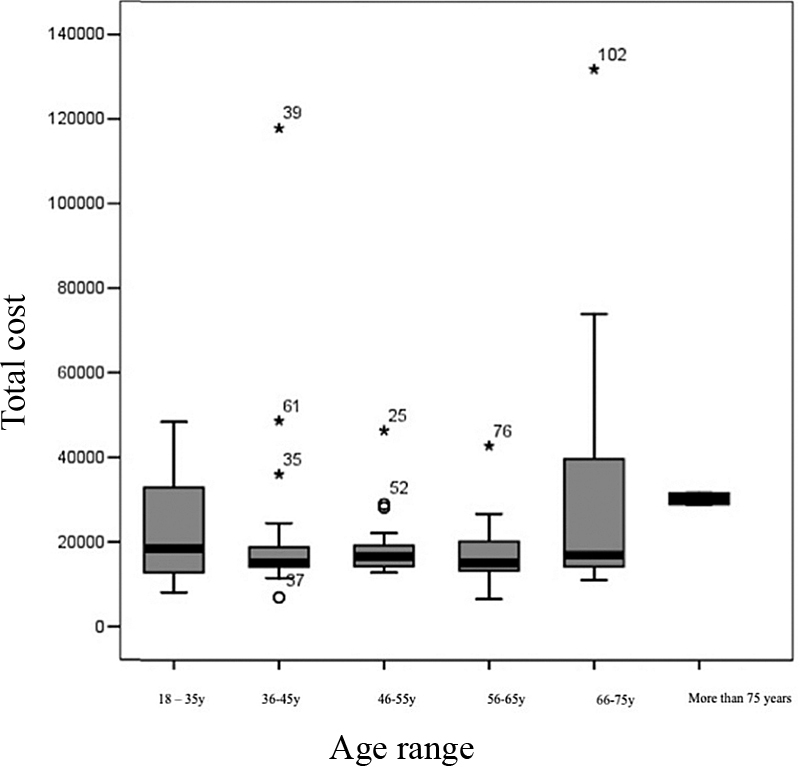
Age range versus total cost.

The mean cost of these patients was R$22,372 (US$4,166). The costs incurred were greater for meningioma patients; however, this association was not statistically significant ( *p* = 0.246). For most surgeries, the costs associated with the operating room ( Figure 2 ) and intensive care unit (ICU) represented the largest proportion of the total cost (29.24 and 24.95%, respectively). Patients aged between 66 and 75 years old had the greatest cost (mean of US$ 5,756.61); however, the association of age with costs incurred was not statistically significant ( *p* = 0.787) ( Figure 3 and Table 1 ).

Eighteen patients (45%) with meningioma had skull base lesions, and the infection rate among them was greater than that in non-skull base patients. Considering the whole group, infections were more common in patients with high-grade glioma (55.5%). In 8 glioma patients, awake craniotomy was performed at a lower cost; however, this association was not statistically significant ( *p* = 0.538). The costs incurred were higher if patients presented with any thromboembolic complications, but this association was not significant ( *p* = 0.308). The main variables analyzed are described in Table 2 .

**Table 2:** Main variables analyzed (age, OR duration in minutes, hospitalization period in days)

Variable	Mean	Median	SD	Minimum	Maximum
Age	51	53	15	14	78
OR occupation (minutes)	489	475	197	75	1242
Hospitalization period (days)	12	7	16	3	123

Abbreviations: OR, operation room; SD, standard deviation.

**Figure 4:**
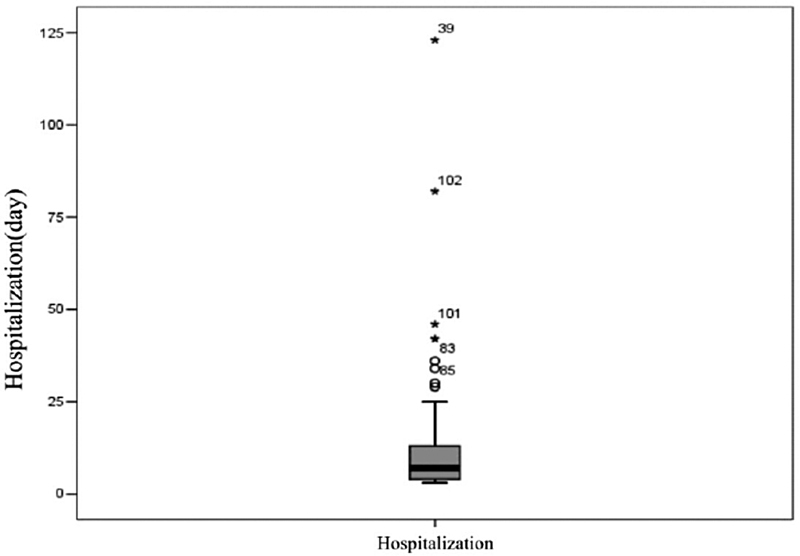
Length of stay (LOS): ICU and neurosurgical ward.

The average hospitalization period was 12 days ( Figure 4 ). The mean ICU period was 3.8 days; however, the costs of ICU were higher than that of neurosurgery infirmary. Nonelective patients had a greater length of stay (LOS). The mortality rate was 12.7%. One patient died after an elective procedure, and 92.3% of deaths occurred after emergency admission. Eighteen patients had some type of infection, and 44% of them died. Table 3 describes the summary of this cohort, considering the presence of thromboembolism, deaths, histology, topography, infections, and age.

**Table 3:** Summary of all patients analyzed: topography, age, thromboembolism, death, infections

Initials	Sex	Age (years old)	Histological analysis	Urgency	Thromboembolic event	Infection	Death
AOA	F	39	MENINGIOMA GRADE I	NO	NO	NO	NO
SEM	F	63	MENINGIOMA GRADE I	YES	NO	NO	NO
ASGS	F	57	MENINGIOMA GRADE I	NO	NO	NO	NO
DLS	M	73	MENINGIOMA GRADE II	YES	NO	MENINGITIS + PNEUMONIA	YES
SRS	F	57	MENINGIOMA GRADE I	NO	NO	MENINIGITIS + PNEUMONIA + SEPSIS	YES
MRSS	F	68	GLIOBLASTOMA	YES	NO	NO	NO
TJS	F	70	MENINGIOMA GRADE I	NO	YES	NO	NO
MJSL	F	60	MENINGIOMA GRADE I	NO	NO	NO	NO
EFS	F	58	MENINGIOMA GRADE I	YES	NO	NO	YES
CRAS	M	48	MENINGIOMA GRADE I	NO	NO	NO	NO
RSD	F	40	MENINGIOMA GRADE I	YES	NO	NO	NO
ESP	F	48	MENINGIOMA GRADE I	NO	NO	NO	NO
JAO	M	63	GLIOBLASTOMA	YES	NO	MENINGITIS	NO
HGA	M	23	ANAPLASTIC OLIGODENDROGLIOMA GRADE III	NO	NO	MENINGITIS	NO
JMSS	M	22	POLIMIXOID ASTROCYTOMA GRADE II	NO	NO	NO	NO
MCL	M	54	GLIOBLASTOMA	NO	NO	NO	NO
JFL	M	60	GLIOBLASTOMA	YES	NO	NO	NO
IDGL	M	26	DIFFUSE ASTROCYTOMA GRADE II	NO	NO	NO	NO
TRCV	M	30	GLIOBLASTOMA	YES	NO	MENINGITIS	YES
TGB	F	36	MENINGIOMA GRADE I	NO	NO	NO	NO
FWFS	M	32	DIFFUSE ASTROCYTOMA GRADE II	NO	NO	NO	NO
JAO	M	63	GLIOBLASTOMA	YES	NO	MENINGITIS	NO
MAS	F	56	MENINGIOMA GRADE I	NO	NO	NO	NO
EDC	M	70	MENINGIOMA GRADE I	YES	NO	NO	YES
NLR	M	54	GLIOBLASTOMA	YES	NO	NO	NO
SPC	F	42	MENINGIOMA GRADE I	NO	NO	NO	NO
ACJ	F	18	ANAPLASTIC ASTROCYTOMA GRADE III	NO	NO	NO	NO
JRTP	M	45	GLIOBLASTOMA	YES	NO	NO	NO
RR	M	39	OLIGODENDROGLIOMA GRADE II	NO	NO	NO	NO
SCGB	F	48	MENINGIOMA GRADE I	NO	NO	NO	NO
JP	F	57	MENINGIOMA GRADE I	YES	NO	NO	NO
CAO	F	46	MENINGIOMA GRADE I	NO	NO	NO	NO
MHSS	F	46	MENINGIOMA GRADE I	YES	NO	NO	NO
ILM	F	58	MENINGIOMA GRADE I	NO	NO	NO	NO
SMS	F	45	MENINGIOMA GRADE I	NO	NO	NO	NO
CBC	M	30	GLIOBLASTOMA	YES	NO	NO	NO
LARS	F	41	GLIOBLASTOMA	YES	NO	NO	NO
BRSP	F	69	MENINGIOMA GRADE I	NO	NO	NO	NO
ELS	M	45	MENINGIOMA GRADE I	NO	NO	MENINGITIS	NO
JFS	M	77	MENINGIOMA GRADE I	NO	NO	NO	NO
JRS	M	66	MENINGIOMA GRADE I	NO	NO	NO	NO
EBS	F	47	MENINGIOMA GRADE I	YES	NO	NO	NO
JCOM	M	36	OLIGODENDROGLIOMA GRADE II	NO	NO	NO	NO
OLF	F	67	MENINGIOMA GRADE I	NO	NO	NO	NO
VAQ	F	64	GLIOBLASTOMA	NO	NO	NO	NO
IAJ	M	45	OLIGODENDROGLIOMA GRADE II	YES	NO	NO	NO
MS	M	56	MENINGIOMA GRADE I	NO	NO	NO	NO
NLR	M	55	GLIOBLASTOMA	NO	NO	NO	NO
JCR	M	60	DIFFUSE ASTROCYTOMA GRADE II	YES	NO	NO	NO
RASP	F	54	GLIOBLASTOMA	YES	YES	NO	NO
MS	M	36	OLIGODENDROGLIOMA GRADE II	YES	NO	NO	NO
ENB	M	47	GLIOBLASTOMA	YES	NO	PNEUMONIA, MENINGITIS, URINARY TRACT INFECTION	YES
COM	M	14	ANAPLASTIC ASTROCYTOMA GRADE III	YES	NO	NO	NO
MO	F	33	OLIGODENDROGLIOMA GRADE III	NO	NO	WOUND INFECTION	NO
MAOS	F	66	MENINGIOMA GRADE I	YES	NO	NO	NO
VAB	M	51	ANAPLASTIC ASTROCYTOMA GRADE III	NO	NO	NO	NO
AMM	M	51	MENINGIOMA GRADE I	YES	NO	NO	NO
LJN	M	68	GLIOBLASTOMA	YES	NO	PNEUMONIA AND URINARY TRACT INFECTION	YES
CLH	F	58	GLIOBLASTOMA	NO	NO	NO	NO
MDN	F	47	MENINGIOMA GRADE I	NO	NO	NO	NO
IFM	M	40	GLIOBLASTOMA	NO	NO	WOUND INFECTION	NO
WMN	M	19	GLIOBLASTOMA	YES	NO	NO	NO
JAFS	M	60	GLIOBLASTOMA	NO	NO	NO	NO
CPU	F	37	GLIOBLASTOMA	NO	NO	NO	NO
JAC	M	73	GLIOBLASTOMA	YES	NO	NO	NO
MAD	F	53	DIFFUSE ASTROCYTOMA GRADE II	NO	NO	NO	NO
MSB	F	44	MENINGIOMA GRADE I	NO	NO	NO	NO
MBD	F	73	GLIOBLASTOMA	YES	NO	NO	NO
MG	M	60	GLIOBLASTOMA	YES	NO	NO	NO
MJSL	F	61	MENINGIOMA GRADE I	YES	NO	WOUND INFECTION	NO
OSN	M	74	GLIOBLASTOMA	YES	NO	NO	NO
NSS	M	54	GLIOBLASTOMA	YES	NO	NO	NO
LJS	M	55	GLIOBLASTOMA	YES	NO	NO	NO
RPC	M	36	OLIGODENGLIOMA GRADE II	YES	NO	NO	NO
FMFS	F	40	GLIOBLASTOMA	YES	NO	NO	NO
FEFG	M	56	GLIOBLASTOMA	YES	NO	PNEUMONIA	YES
MRS	F	40	ANAPLASTIC ASTROCYTOMA GRADE III	YES	NO	NO	NO
RAIS	F	59	ANAPLASTIC OLIGODENDROGLIOMA GRADE III	YES	NO	NO	NO
MLFA	F	64	MENINGIOMA GRADE I	YES	NO	NO	YES
ACS	M	51	GLIOBLASTOMA	YES	NO	NO	NO
GVM	M	46	GLIOBLASTOMA	NO	NO	NO	NO
WOS	F	78	MENINGIOMA GRADE I	YES	NO	PNEUMONIA	YES
JVS	M	71	GLIOBLASTOMA	YES	NO	NO	YES
JSS	M	49	GLIOBLASTOMA	NO	NO	NO	NO
MAS	F	71	MENINGIOMA GRADE I	YES	YES	NO	NO
AOS	F	32	GLIOBLASTOMA	YES	NO	NO	NO
MLAS	F	58	GLIOBLASTOMA	YES	NO	NO	NO
MRSS	F	68	GLIOBLASTOMA	NO	NO	NO	NO
MMS	M	70	GLIOBLASTOMA	NO	NO	NO	NO
CRM	F	42	OLIGODENDROGLIOMA GRADE II	NO	NO	NO	NO
DAL	M	56	GLIOBLASTOMA	YES	NO	NO	NO
AS	M	26	MENINGIOMA GRADE II	YES	NO	NO	NO
RSMJ	M	22	GLIOBLASTOMA	NO	NO	NO	NO
EM	M	49	GLIOBLASTOMA	YES	NO	NO	YES
VMO	F	68	GLIOBLASTOMA	YES	NO	PNEUMONIA	NO
CBC	F	48	MENINGIOMA GRADE I	YES	NO	NO	NO
JJN	F	68	GLIOBLASTOMA	YES	NO	PNEUMONIA	NO
FO	M	38	ANAPLASTIC OLIGODENGLIOMA GRADE III	NO	NO	NO	NO
MMS	M	53	DIFFUSE ASTROCYTOMA GRADE II	NO	NO	WOUND INFECTION	NO
ACOS	F	16	GANGLIOGLIOMA GRADE I	YES	NO	NO	NO
RQS	M	26	MENINGIOMA GRADE I	YES	NO	MENINGITIS	NO
ACPF	M	67	MENINGIOMA GRADE I	YES	NO	NO	YES
NSS	M	54	GLIOBLASTOMA	YES	NO	NO	NO

## DISCUSSION

The treatment priorities of neuro-oncological patients include quality of life (QOL) and overall survival (OS). High neurosurgical costs can impact public health, even if patients have medical insurance. Comparing the health costs incurred in different countries can help to better understand the deficits in healthcare and how to improve them. 11

Documenting costs in neuro-oncology is important for resource allocation, healthcare sector planning, and adoption of cost-saving interventions, and for clinical research aimed to improve the QOL of patients. 12 The cost of neurosurgical intervention is the sum of the direct and indirect costs. Direct costs can be attributed to a specific service or procedure, whereas indirect costs cannot. Identifying patient groups or interventions associated with higher treatment costs may be beneficial in promoting efforts to decrease the overall financial burden. Strategies to reduce cost may require different approaches depending on the type of procedure. 13 14 Therefore, we did not compare the costs of microsurgery and endoscopy in the present evaluation – each procedure had several advantages. Providing a cheaper but effective treatment not only improves the economy, but also the QOL and OS for the patient.

The mean cost of treating these patients was R$22,372 (US$4,166), which was lower than the cost calculated in other studies. Goel et al. 14 calculated a mean cost of U$10,042 for craniotomy after analyzing 21 studies in 13 countries. 15 However, most of these studies considered private, and not public, institutions in developing countries.

The time spent in the operating room was the most important variable that affected the mean total cost ( Figure 2 ). As part of the neurosurgical and anesthesiology residency program, this association could be explained by the participation of residents in training.

Length of stay is a useful measure of healthcare quality. Increased LOS is associated with higher healthcare costs. 16 17 A postoperative LOS of ≥ 14 days has been associated with an increased frequency of surgical site infections (SSIs) and a rise in healthcare costs of up to 300%. 15 In the present study, the mean LOS was 12 days ( Figure 4 ). For elective procedures, the LOS was 3 days. Several patients admitted to the emergency department were not operated upon immediately, as was done in elective procedures in which patients were at home and admitted only when appropriately prepared for surgery. With prolonged LOS, the rate of complications such as infections and thrombosis increased. 18 The present study only evaluated adult patients; the characteristics for analysis in children are different. 16

The development of safe and effective standards for postoperative care has emerged as a key factor in improving patient outcomes and reducing costs. 7 19 In 1994, Engelmann and colleagues introduced the concept of fast-track surgery to optimize postoperative recovery. 18 As a precautionary measure, many neurosurgical centers still adopt postoperative care with a mean of 4 days after craniotomy, even in cases with no perioperative complications. As we observed in the present study, a longer hospitalization period is associated with greater costs, especially in the ICU ( Figure 4 ).

Shorter hospital LOS has been associated with decreased rates of complications, fewer hospital-acquired infections, and lower costs. 18 19 Due to concerns regarding postoperative complications, neurosurgeons could be hesitant in discharging patients on the same day or 1 day after craniotomy. The most severe postoperative sequelae occur within 24 hours after surgery. Observing the patients overnight can limit the number of complications. With the evolution of surgical technology, instrumentation, monitoring techniques, and increased proficiency in anesthesia, patients are now receiving improved perioperative care with shorter operative duration, shorter recovery, and faster discharge.

In the present study, the LOS in the ICU was the second biggest factor responsible for increased costs. A recent study revealed that the cost differential between the ICU and neurotransitional care units is US$1,504 per day. 20 Some postoperative brain tumor patients could be monitored in a semi-intensive unit, which could decrease the costs. 13 If health professionals are trained for a more dynamic and effective patient approach, discharge for patients could be earlier and safer. 20 21 22

The present study also revealed that emergency procedures are related to a longer hospitalization period, infections, deaths, and higher total incurred costs. In the present study, the patients did not undergo appropriate surgical preparation, and several of the patients had uncontrolled arterial hypertension, diabetes mellitus, and obesity, and were smokers or malnourished. In rare cases, the brain tumors required emergency surgery.

Unfortunately, five billion people worldwide do not have access to safe, affordable surgical and anesthetic care when needed, 1 and only receive health assistance when they are severely ill, especially in low-income and lower-middle-income countries. Better patient preparation and fewer emergency procedures are reasonable options for cost reduction.

Although the association between awake-state surgery and smaller costs was not statistically significant, it was biased due to a small number of awake procedures in the present study. 23 Several studies have described a shorter LOS in patients who undergo awake-state craniotomies. 11 16 24 25 26 27 For selected cases, awake neurosurgery improved functional outcomes characterized by a small LOS. The involvement of a multidisciplinary team is required for awake-state surgery.

In the postoperative period, infections and thromboembolic events can cause higher morbidity, mortality, and costs. 27 Surgical site infection (SSI) incidence in neurosurgery is low, and most readmissions occur within 30 days. 27 28 29 Broad-spectrum antibiotics are expensive; therefore, careful surgical preparation should be encouraged. 30 31 Regarding the histological type of tumors and the costs involved, meningiomas incurred a greater cost; however, this association was not statistically significant. For skull base meningiomas, the procedures were longer, and the recovery was slower. 32 33 Furthermore, the infection rate was greater in skull base meningiomas than that in non-skull base meningiomas. In the meningioma group, skull base localization (45%) was associated with greater costs.

Regarding gliomas, low grade glioma (LLG) predominates in younger patients, ( Figures 1 and 3 ) who have faster recovery and shorter hospitalization, which did not happen to high grade glioma (HGG), especially glioblastoma (GBM). 34 35 36 In the present cost evaluation, glioblastoma emerged as the most common tumor, with an incidence of 22.75% ( Figure 1 ). GBM is an aggressive, high-grade tumor associated with a significant clinical burden. 33 Nearly half of the primary malignant brain tumors in adults are GBMs. According to the Central Brain Tumor Registry of the United States, the average annual age-adjusted incidence of GBM is 3.2 cases per 100,000 people.

Several studies have previously calculated the specific costs of low-grade gliomas and GBM; however, they included adjuvant therapy in the total cost, which was not performed on the patients included in the present study. 37 38 39 40 In the USA, a patient remains in one center for the entire treatment, which allows a better evaluation of the data. 40 Unfortunately, in Brazil, patients need to perform each part of their treatment in one sector; hence, the records are not uniform. For example, there is no radiotherapy or radiosurgery services at the institution where the present study was conducted.

Health economics of GBM is a subject of rising interest, but there is only limited knowledge regarding cost-effectiveness and other economic aspects of different therapies for recurrent GBM. 34 38 40

A high proportion of patients with GBM require emergency department visits (32%) and hospitalizations (28%) in the 6 months following the diagnosis, which is indicative of the substantial healthcare resource burden associated with GBM. 38 Emergency neurosurgical approaches are also commonly required, as documented by the present study. 27 We observed that the mortality rate was higher in the emergency group (92.3%). Only one elective patient died in the present study – an elderly female patient with several comorbidities, with an initial Karnofsky Performance Scale score of 40 and a giant olfactory groove meningioma. The patient died of a refractory septic shock due to pneumonia. Adjuvant therapy was not discussed in the present study, but it adds to the increased treatment costs.

In addition, survivors of brain tumors experience countless socioeconomic impacts, especially if the tumor is malignant. Even in a population of stable, high-functioning patients, financial burden and workforce morbidity was ubiquitous across all tumor subtypes, treatment paradigms, and income levels. 39 40 Patients also had an increased risk of anxiety, depression, and neurocognitive deficits. 39 41

Implementing digital referrals to a multidisciplinary neuro-oncological triage panel may help reduce costs. This approach is feasible and would facilitate swift referrals that are tailored for the patients at costs and time investment that are comparable to standard referrals. 38

## Limitations

The present study has several limitations. The results of a retrospective cost analysis from a single-center, mixed-case index academic practice may not apply to all centers, depending on the proportion of cases. In the Brazilian unified public health system (SUS, in the Portuguese acronym), the availability of molecular markers for better characterization is rare. The present study did not consider the cost of neuro-oncological patients who did not undergo neurosurgery.

In conclusion, cost analysis in the neuro-oncological field has recently gained an increased research interest. Neurosurgical procedures are highly driven by technology and often require extensive workup. They can result in prolonged hospitalizations because of the morbidity of neurologic injury, even when the procedures are performed without surgical complications. Optimization of resources, especially when attempting to reduce the operating room (OR) time in minutes, LOS, and postoperative infections, could be an excellent alternative to help in reducing the costs incurred and in achieving better outcomes.
